# The Optical Spectrum of Au_2_^+^

**DOI:** 10.1002/anie.202011337

**Published:** 2020-10-12

**Authors:** Marko Förstel, Kai Mario Pollow, Karim Saroukh, Este Ainun Najib, Roland Mitric, Otto Dopfer

**Affiliations:** ^1^ Technische Universität Berlin Hardenbergstr. 36 10623 Berlin Germany; ^2^ Julius-Maximilians-Universität Würzburg Institut für Physikalische und Theoretische Chemie Emil-Fischer-Str. 42 97074 Würzburg Germany

**Keywords:** cations, electronic structure calculations, gold, optical spectroscopy, photodissociation

## Abstract

The electronic structure of the Au_2_^+^ cation is essential for understanding its catalytic activity. We present the optical spectrum of mass‐selected Au_2_^+^ measured via photodissociation spectroscopy. Two vibrationally resolved band systems are observed in the 290–450 nm range (at ca. 440 and ca. 325 nm), which both exhibit rather irregular structure indicative of strong vibronic and spin‐orbit coupling. The experimental spectra are compared to high‐level quantum‐chemical calculations at the CASSCF‐MRCI level including spin‐orbit coupling. The results demonstrate that the understanding of the electronic structure of this simple, seemingly H_2_^+^‐like diatomic molecular ion strictly requires multireference and relativistic treatment including spin‐orbit effects. The calculations reveal that multiple electronic states contribute to each respective band system. It is shown that popular DFT methods completely fail to describe the complex vibronic pattern of this fundamental diatomic cation.

## Graphical abstract

The optical spectrum of the prototypical diatomic Au_2_^+^ cation is presented in high quality. The measured spectrum of this seemingly simple H_2_^+^‐like cation cannot be explained by standard TD‐DFT methods but requires relativistic multireference treatment with spin‐orbit coupling.

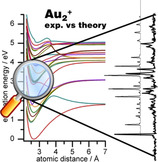

At first glance, the unpaired electron in the bonding σ(s) orbital of Au_2_^+^ lets this dimer appear to be as simple as H_2_^+^. However, large relativistic effects,^[1]^ spin‐orbit (SO) coupling, ω‐ω instead of L‐S coupling,^[2]^ and d‐orbital contributions to bonding^[3]^ make this system much more challenging to understand. Nevertheless, a detailed quantitative picture of the electronic structure of this simple diatomic cation is a necessary requirement for understanding more complex phenomena and properties including, but not limited to, the structure of gold clusters,^[4]^ the bond activation and catalytic properties of gold clusters and nanoparticles,[^4d^, ^4e^, ^4f^, ^5^] and their medical and biological applications.^[6]^

We present the first optical spectrum of Au_2_^+^ measured in the range from 300 to 700 nm (1.77–4.13 eV) in a quadrupole–reflectron time‐of‐flight tandem mass spectrometer coupled to a cooled laser vaporization source. This recently improved setup allows for hitherto unobtainable sensitivity and spectral resolution for optical spectroscopy of ultra‐dilute targets.^[7]^ We observe two band systems that provide a detailed probe of the excited‐state potential energy surfaces (PESs) of Au_2_^+^ by comparison to high‐level complete‐active‐space self‐consistent‐field multireference configuration interaction (CASSCF‐MRCI) calculations including SO coupling.

The neutral Au_2_ dimer has been studied in more detail than its cation. Experimentally, the bond dissociation energy was determined as *D*_0_=2.290±0.008 eV,^[8]^ with theoretical values being slightly lower.^[9]^ The ionization energy is in the order of IE=9.2±0.2 eV.[^8b^, ^9a^, ^9b^, ^10^] Electronic band systems of Au_2_ were observed in rare gas matrices^[11]^ and the gas phase (some rotationally resolved)[^8b^, ^12^] and were discussed theoretically.[^9c^, ^13^] The ground state of the Au_2_^+^ cation is ^2^Σ_g_^+^, with the unpaired electron largely localized in the σ(s) orbital^[14]^ and *D*_0_=2.20±0.21 eV.^[5i]^ Estimates based on *D*_0_ and IE of Au_2_ result in *D*_0_=2.32±0.21^[8b]^ and 2.36±0.10 eV for Au_2_^+^.^[10]^ Owen et al. report calculated values of *D*_0_=2.09 (B3LYP/def2), 2.02 (CCSD(T)/def2), 1.86 (M06‐2X/def2), and 2.04 eV (CCSD(T)‐F12/cc‐pVTZ‐pp).^[14]^ Earlier work reported *D*_0_=1.75 eV using a size‐extensive self‐consistent field based on a modified coupled‐pair functional approach^[9b]^ and *D*_0_=1.79 eV using a complete active space followed by a multi‐reference singles+doubles CI method.^[9a]^ Owen et al. also calculated relative energies, binding energies, and bond lengths of four low lying excited states (ESs) of Au_2_^+^ (^2^Σ_u_^−^, ^2^Π_g_, ^2^Δ_u_, ^4^Σ_g_^+^).^[14]^

Despite the limited knowledge for bare Au_2_^+^, many studies deal with its complexes with molecular ligands, especially in the context of bond activation and catalysis. Among these systems are Au_2_^+^O_2_(C_2_H_4_)_*n*_ and Au_2_^+^(C_2_H_4_)_*n*_, in which activation of the C=C and O−O bonds was observed.[^5a^, ^5b^, ^5c^] The Au_2_^+^(CH_4_)_*n*_ complexes show C−H bond dissociation and/or activation[^5d^, ^5e^] and were reported to form ethylene (C_2_H_4_) catalytically.[^5c^, ^15^] These results were cast into doubt, suggesting that either an ES of Au_2_^+^ causes the experimental results^[5g]^ or that C−H activation occurs without H_2_ elimination.[^5h^, ^5i^] The Au_2_^+^(CO)_*n*_ system was discussed also and both, dissociation of CO and bonding of CO to the Au_2_^+^ dimer, were reported.[^5k^, ^5l^] In all these Au_2_^+^‐ligand complexes, many questions remain open and most of the experimental results require a reliable theoretical method to allow for meaningful interpretation of the data concerning the electronic structure of bare Au_2_^+^. With our experimental results on Au_2_^+^, we provide a benchmark for such calculations and our theoretical work points at the limits and requirements of those. The few existing calculations do not reproduce our experimental results.^[16]^

The optical spectrum of Au_2_^+^ is obtained by electronic photodissociation (EPD), a variant of highly‐sensitive indirect action spectroscopy to infer the absorption spectrum. Due to the low Au_2_^+^ concentration, direct photoabsorption cannot be employed because of insufficient sensitivity. Although the EPD cross section only represents a lower limit of the absolute photoabsorption cross section, it is a very good approximation for cases where fluorescence is weak.^[17]^ The relatively low oscillator strengths for the observed transitions of Au_2_^+^ correspond to long fluorescence lifetimes which makes dissociation the dominant relaxation channel. We avoid the popular approach of messenger‐tagging, in which a weakly bonded ligand (e.g., He or H_2_/D_2_) is attached to the target ion to facilitate efficient photodissociation, because even such a small external perturbation can significantly disturb the electronic and thus optical properties of Au_2_^+^.^[17]^ Instead we directly dissociate bare Au_2_^+^ by single‐photon absorption into Au^+^ and Au (Figure S1 in the Supporting Information (SI)). This condition means that we can only observe optically accessible states above the lowest dissociation threshold of Au_2_^+^ reported as *D*_0_=2.21±0.21 eV.^[14]^ Experimental details are described in the SI.

In the EPD spectrum measured in the 300–700 nm range (Figure S2 in the SI), we observe two band systems as shown in Figures 1 and 2 (Table S1 and S2 in the SI). The first band system (BS1) measured at *T*=150 K starts with a small peak at 444.05 nm (a1, 22 520 cm^−1^, 2.79 eV) followed by the strongest peak of the system at 440.5 nm (a2, 22 701.5 cm^−1^, 2.81 eV). Several small peaks follow, the last one of which is seen at 422.21 nm (a15, 23 685 cm^−1^, 2.94 eV). The peaks have a FWHM of 8–10 cm^−1^, which is in the order of the available instrumental resolution given by the laser bandwidth and the unresolved rotational contour. Interestingly, a clear vibrational progression, as observed for the neutral Au_2_ dimer^[8b]^ or the Au_4_^+^ radical cation^[7a]^ cannot be identified in the rather irregular appearance of the spectrum. The spacing between a6 and a10 and between a6 and a2 (182 and 2×182 cm^−1^) are indicative of a progression with 182 cm^−1^. This frequency, in turn, allows a crude estimate of the Au‐Au bond length of the corresponding excited state as 2.48 Å, which agrees well with previous calculations assuming that an antibonding electron is promoted to a bonding orbital.^[14]^ Another discernable pattern is the equal spacing of 40.5 cm^−1^ between a9, a10 and a11. Apart from that, we can only note that BS1 cannot be explained easily by assuming only a single underlying excited state of a diatomic ion. Also included in Figure 1 is an EPD spectrum taken at *T*=300 K with the goal to identify potential hot bands. The spectrum looks more congested than the spectrum at *T*=150 K, and a 30 cm^−1^ broad shoulder appears below a1. Almost unchanged intensity is seen for peaks a1, a2, a8 and a11. This observation is taken as an indication that a1 represents a band origin.

**Figure 1:**
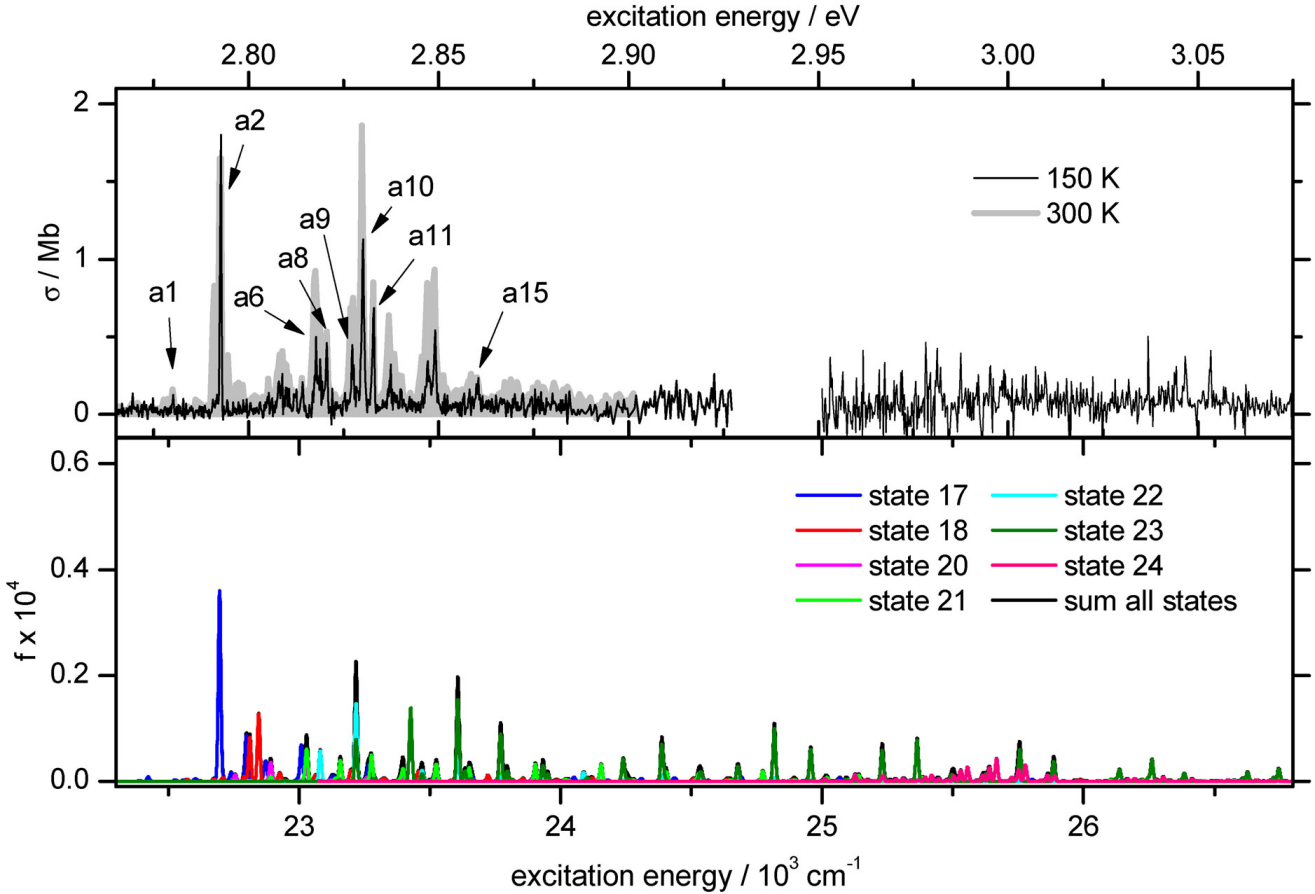
EPD spectrum of band system 1 (BS1) of Au_2_^+^ measured at a nozzle temperature of *T*=150 (black) and 300 K (gray area) in comparison to the calculated transitions of all corresponding excited states in this energy range (bottom panel). The calculated spectra are shifted by −929 cm^−1^ and folded with a Gaussian line profile with 8 cm^−1^ FWHM. Experimental peaks (Table S1 in the SI) discussed in the text are labeled. The different colors in the calculated spectrum refer to transitions from the respected ES as indicated in the legend. The gap in the experimental trace is due to insufficient laser power in that range.

The second band system (BS2, Figure 2, *T*=120 K) observed in the range 29 250–32 500 cm^−1^ (342–308 nm, 3.63–4.03 eV) exhibits about 60 discernable peaks (b1‐b57) with widths down to 10 cm^−1^, which is again limited by the instrumental resolution. BS2 is even more congested and irregular than BS1 and again, we cannot find a simple pattern as expected for a transition into a single bound excited state with a Morse‐like potential. BS2 starts with a set of three peaks (b1‐b3) at 29 308, 29 425 and 29 775 cm^−1^ (3.63, 3.65 and 3.69 eV). A region of several small peaks with low intensity follows until, starting with peak b8 at 30 257 cm^−1^, the intensity of the following peaks increases. The strongest peak in BS2 at 30 931 cm^−1^ (b25, 3.83 eV) has a cross section of 14.5 Mb, and thus is about eight times stronger than the most intense band in BS1 (a2). The overall integrated intensity of BS2 is about 20 times that of BS1. Several regions of closely spaced peaks are seen. The last clearly discernable peak is observed at 32 289 cm^−1^ (b57, 4.00 eV).

**Figure 2:**
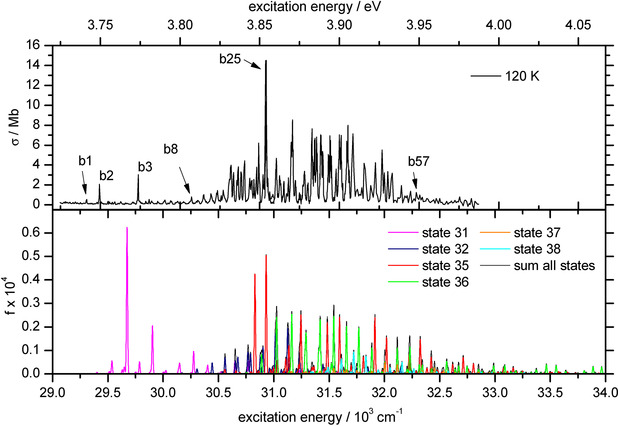
EPD spectrum of band system 2 (BS2) of Au_2_^+^ measured at a nozzle temperature of *T*=120 K and the calculated transitions of all contributing excited states in this energy range (bottom panel). The calculated spectra are shifted by +1678 cm^−1^ and folded with a Gaussian line profile with 10 cm^−1^ FWHM. Experimental peaks (Table S2 in the SI) discussed in the text are labeled. The different colors in the calculated spectrum refer to transitions from the respected ES as indicated in the legend.

A more quantitative understanding of the EPD spectra is possible by comparing the results to the high‐level quantum chemical calculations combined with the simulations of vibronic spectra. To this end, we calculate the electronic states of Au_2_^+^ by employing the state‐averaged CASSCF method (SA‐CASSCF) followed by internally contracted multireference configuration interaction (MRCI) calculations^[18]^ as implemented in the MOLPRO program package.^[19]^ For the gold atom, the relativistic 19‐electron effective core potential (MDF60) developed by Figgen et al.^[20]^ combined with the (12s12p9d3f2g)/[6s6p4d3f2g] contracted atomic basis set is used.^[20]^ SO effects are included by diagonalizing the SO Hamiltonian in the basis of MRCI eigenstates using the MDF60 SO‐pseudopotentials to represent the SO coupling operator. The active space for the SA‐CASSCF calculations consists of the 10 orbitals derived from the atomic 5d orbitals and two valence orbitals derived from the 6s atomic orbitals. The 5s and 5p core orbitals are kept frozen. The potential energy curves and the transition dipole moment components for all electronic states are pre‐calculated on a fine grid extending from 2.1 to 7.1 Å. Cubic spline interpolation is used to obtain a continuous representation of the curves. The vibrational eigenstates for each adiabatic electronic state are calculated numerically on a grid containing 512 points by using the Fourier representation of the kinetic energy operator. Vibrationally resolved electronic spectra are simulated starting from a Boltzmann population of the initial vibrational states in the ground electronic state at different temperatures. In order to calculate spectral line intensities the transition dipole moments between all pairs of initial and final states are calculated using numerical integration and taking into account the full R‐dependence of the electronic transition dipole moments. For comparison, we also applied time‐dependent density functional theory (TD‐DFT) calculations at the CAM‐B3LYP/aug‐cc‐pVTZ level.^[21]^

The calculated PESs at different levels of theory for the ground and excited electronic states of Au_2_^+^ are shown in Figure 3. The equilibrium atomic distance in the ^2^Σ_g_^+^ ground state (GS) is calculated as *r*_e_=2.64 Å at the MRCI‐SO level with a dissociation energy of *D*_e_=1.84 eV, calculated as the difference of the energies at *r*_e_ and *r*=7.15 Å. This energy is lower than the recently calculated values and the experimental value of *D*_0_=2.21±0.21 eV.^[14]^ The dissociation energy calculated at the CCSD(T) level has a value of *D*_e_=1.98 eV (Figure S4 in the SI). By comparing the experimental dissociation energy of Au_2_, *D*_0_=2.290±0.008 eV,^[8]^ to the one of Au_2_^+^ and considering that Au_2_^+^ has one less bonding electron than Au_2_, one would expect that *D*_0_ lies at the low energy end of the reported experimental range, as predicted by the CCSD(T) level.

**Figure 3:**
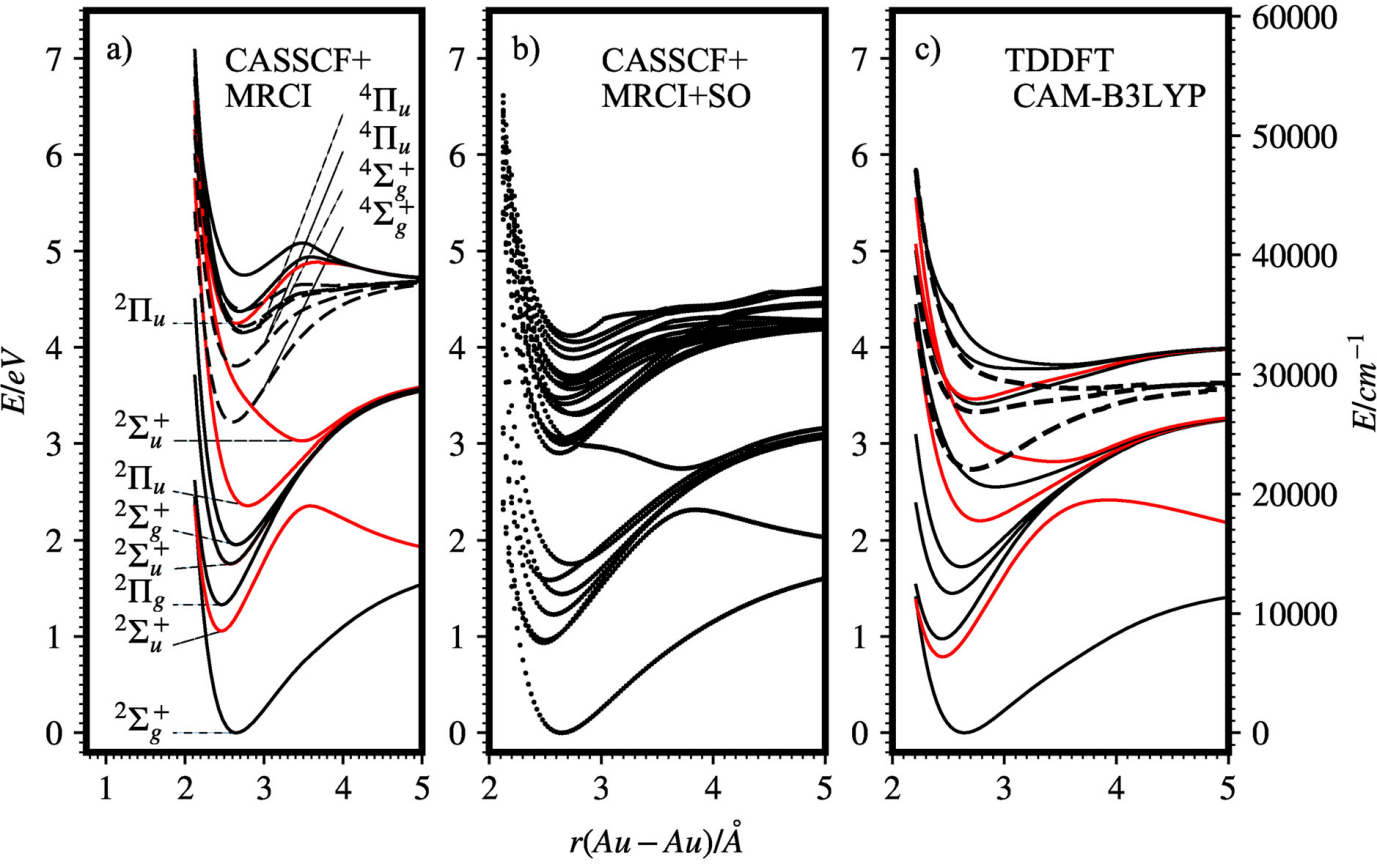
Calculated potential energy surfaces (PESs) of Au_2_^+^ at different levels of theory. Red curves in (a) and (c) indicate states with nonzero oscillator strength. Dashed curves in (a) and (c) indicate quartet states.

While the calculated GS PESs are very similar for the three different theoretical models, we find large differences in the ES PESs (Figure 3). The ES PESs below 3 eV of the TD‐DFT and the non‐SO‐coupled MRCI calculations (panels a) and c) in Figure 3) are comparable in relative position (for lower ESs) and shape of the PESs of the corresponding states. However, the absolute energies calculated with TD‐DFT are about 0.2–0.5 eV lower than those calculated using MRCI with the difference increasing with higher lying states. At higher energies, the differences increase significantly and the relative ordering of states changes. The second optically allowed transition calculated with non‐SO‐coupled MRCI (^2^Π_u_) has its minimum 2.4 eV above the GS. The corresponding state minimum calculated with TD‐DFT occurs at 2.2 eV. The lowest lying quartet state ^4^Σ_g_^+^ is found at 3.2 eV (2.7 eV in case of TD‐DFT) above the GS. Optically allowed transitions to the ^2^Σ_u_^+^, ^2^Π_u_ and ^2^Π_u_ states calculated without SO coupling have vertical excitation energies of 1.2, 2.4 and 3.7 eV, respectively (0.97, 1.98 and 3.48 eV for TD‐DFT). If the two transitions ^2^Π_u_ ← ^2^Σ_g_^+^ and ^2^Σ_u_ ← ^2^Σ_g_^+^ were the observed ones (^2^Σ_u_^+^ ← ^2^Σ_g_^+^ is well below *D*_0_), then the calculations are 0.4 and −0.4 eV off from the experiment, respectively. The TD‐DFT calculated oscillator strengths and the simulated Franck–Condon progressions also do not resemble the observed spectrum (Figure S3 in the SI).

After inclusion of SO coupling, the picture changes drastically (Figure 3, panel b)). The first five PESs consisting of several groups of ESs move closer together in energy. We find eight closely spaced ESs between 2.8 and 3.2 eV that are derived from the optically allowed states with ^2^Σ_u_^+^ and ^2^Π_u_ symmetry mixed with the lowest quartet states with ^4^Σ_g_^+^ symmetry via SO coupling. In the range of BS2, we find again eight closely spaced excited state manifolds derived from coupling quartet states with ^4^Σ_g_^+^ and ^4^Π_u_ symmetry with all close lying doublet states. The lower lying states are not accessible by single‐photon EPD because they are all below *D*_0_. (Although some vibrationally high lying states may dissociate, the FC factors are probably low because the GS and fifth ES PESs are very similar.)

To allow for a more detailed comparison with the experiment, we show the calculated line positions and intensities in Figures 1 and 2. The calculated transitions in the range of BS1 are shifted by −929 cm^−1^ to align the peak with highest calculated strength to peak a2. The different colors represent the different ESs in that range. Only states that contribute significantly to intensity in that range are plotted. The calculated spectrum has a width of about 0.25 eV, with the highest intensity peaks occurring at lower energies. States numbered 17, 18, 21, 22 and 23 are responsible for most of the intensity. While the overall shape of the calculated spectrum resembles the experimental one, we cannot assign individual peaks easily. Peak a2 could be a transition into state 17 and a10 may be a transition into state 22 or 23. However, very small changes in the PESs lead to large changes in relative intensity of the calculated peaks. The calculated intensity above 24 000 cm^−1^ stems mostly from transitions into state 23. The discrepancy of the calculated and the experimental spectrum above 24 000 cm^−1^ could be caused by an overestimation of the oscillator strength and the Franck–Condon factor of state 23.

Plotted in Figure 2 are the calculated intensities of the transitions in the energy range of BS2. They are shifted by +1628 cm^−1^ (matching the position of the strongest peak from state 35 to the position of b25) and plotted color‐coded by corresponding ES. Such a difference between theory and experiment is common for systems containing metal atoms even at the employed high level of theory.^[22]^ Again, a one‐to‐one peak assignment cannot be done. However, the overall contour and peak density resemble the experimental spectrum. Even though the intensities are not reproduced, we can assume that b1‐b3 are transitions into state 31 due to the unique position at the low energy end of BS2. At least some part of b25 stems from a transition into state 35 and the range of closely spaced peaks with similar intensity between 31 250 and 31 750 cm^−1^ (b35‐b47) is caused by transitions into states 35 and 36. Similar to BS1, we have some calculated intensity at the high energy side of the spectrum that is not observed experimentally. In summary, we can say that neither the TD‐DFT nor the non‐SO‐coupled MRCI calculations can predict the experimental spectrum of this seemingly simple diatomic ion. It is thus crucial to consider SO coupling to predict the position and shape of the observed band systems.

In conclusion, we present herein the first absorption spectrum of isolated Au_2_^+^ and compare the results with several theoretical models. The spectrum does not show a simple vibronic structure with a clear regular vibrational progression of individual excited states as observed for example for Au_2_, which is a closed‐shell system.[^8b^, ^12^] Instead, we observe two band systems with seemingly irregular peak positions and intensities. We show that the spectrum can be explained by strong SO coupling and resulting shifts of the coupled excited state surfaces. The observed band systems are caused by several closely spaced excited states which make a quantitative understanding very challenging. In fact, while we can reproduce spectral shape and position and possibly some of the many observed peaks, the method is still not accurate enough to enable an assignment of specific peaks to vibrational transitions. Nonetheless, the electronic structure seems now well established, and may provide in the future deeper insight into the catalytic properties of Au_2_^+^. The presented experimental high‐resolution data will allow for further improving (fine‐tuning) the PESs of Au_2_^+^ by minor adjustments using morphing and/or fitting algorithms which eventually will allow to fully assign the experimental vibronic peaks. Our results demonstrate that this simple diatomic ion with a single σ(s) electron in the HOMO has a complex electronic structure that requires relativistic treatment and consideration of SO coupling.

## Conflict of interest

The authors declare no conflict of interest.

## Supporting information

As a service to our authors and readers, this journal provides supporting information supplied by the authors. Such materials are peer reviewed and may be re‐organized for online delivery, but are not copy‐edited or typeset. Technical support issues arising from supporting information (other than missing files) should be addressed to the authors.

Supplementary
