# Supplementary material for: The Optical Spectrum of Au2 +
Source: Angew Chem Int Ed Engl. 2020 Oct 12;59(48):21403–8. doi: 10.1002/anie.202011337 (PMC7756737; doi:10.1002/anie.202011337)
Supplement: Supplementary file 1 — Supplementary [file ANIE-59-21403-s001.pdf]

Angewandte Chemie
Eine Zeitschrift der Gesellschaft Deutscher Chemiker

Supporting Information

The Optical Spectrum of Au_2^+

Marko Förstel, Kai Mario Pollow, Karim Saroukh, Este Ainun Najib, Roland Mitric,* and Otto Dopfer**

anie_202011337_sm_miscellaneous_information.pdf

CONTENT

Experimental details

Figure S1: Explanation for On/Off signal processing

Figure S2: All scans experimental traces

Figure S3: MRCI-CASSCF versus MRCI-CASSCF-SO and TD-DFT FC simulation

Figure S4: CSSD(T) ground state potential for Au_2^+

Table S1: Peak positions and intensities for BS1

Table S2: Peak positions and intensities for BS2

References

Experimental details

The EPD spectra are recorded in a recently described laser-vaporization tandem mass spectrometer setup.[1-2] Au_2^+ ions are generated via laser vaporization from a turning and translating Au rod using a focused laser pulse at 355 nm (2 mJ) from a Q-switched Nd:YAG laser operated at 20 Hz. The generated plasma is expanded through a LN_2-cooled and temperature-controlled conical nozzle (T=77-300 K) with the help of a He carrier gas pulse from a Parker General Valve at a backing pressure of 8 bar. The opening time of the valve is 300 µs and limited by the maximum permissible pressure of the expansion chamber of about 10^{-4} mbar. After passing a conical skimmer, the clusters enter a quadrupole mass spectrometer (QMS) with a maximum mass resolving power of $\Delta m/m$=1000 that is set to accept only ions with m/z 394. The mass-separated clusters then enter the extraction region of a reflectron time-of-flight mass spectrometer (ReTOF-MS). Here, every second ion bunch (laser-on) is overlapped with a laser pulse from a Nd:YAG pumped optical parametric oscillator (OPO, 10 Hz, 5-10 cm^{-1} bandwidth, 192-2750 nm tuning range, 0.5-150 mJ/pulse) that is timed to hit the ions 2 µs before applying the high voltage extraction pulse of the ReTOF-MS. The resulting ReTOF-MS spectra are sorted into laser-on and laser-off spectra and then summed up for each excitation laser wavelength. The laser power is recorded for each wavelength using a calibrated pyroelecetric power meter (Gentec). The laser has a bandwidth of about 5 cm^{-1} above 400 nm and about 10 cm^{-1} below that. Absolute wavelength uncertainty is 10 cm^{-1}. The laser power was kept below 5 mJ per pulse to avoid two-photon processes. Typical resulting mass spectra are shown in Figure S1. Here, the laser-on signal includes both, parent and fragment ions and is shown unscaled. The barely visible fragment peak is seen more clearly in the magnified difference spectrum (on-off)x50.

The reported photodissociation cross section is derived using a modified Lambert-Beer law as described in references 2-3, thus accounting for variations in the photon flux and the parent ion intensity. The overlap factor of laser beam and ion beam has been determined in a previous experiment and is in the order of α=0.6±0.3. The fragment signal strength is obtained by integrating the respective mass peaks in the laser-on spectrum and the parent strength stems from the laser-off mass spectrum. The absolute uncertainty in the cross section strongly depends on the uncertainty in the overlap factor. For the first peak at 440.5 nm of BS1, the dissociation cross section is σ=(2 +2/-1) Mb. The obtained photodissociation cross section represents a lower limit for the absolute photoabsorption cross section.

The presented EPD spectra are measured at three different nozzle temperatures. BS1 observed in the 22000-24000 cm^{-1} range is recorded at 150 and 300 K, while BS2 is measured at 120 K. In principle, we want to measure the EPD spectra under as cold as possible conditions to reduce spectral congestion arising from hot bands. However, the observed parent signal intensity also decreases with decreasing nozzle temperature and the chosen temperature is thus a compromise between acceptable parent ion intensity and lowest possible temperature. Band system BS2 can thus be measured at a lower temperature than BS1 because it has a roughly twenty times higher cross section.

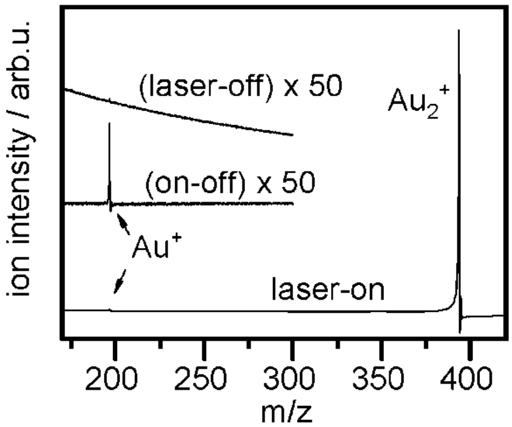

Figure S1. Integrated mass spectra of all laser-on spectra correpsonding to BS1 measured at T=150 K. Included are the magnified traces of laser-off and (laser-on minus laser-off) in the Au^+ mass range. The slope in the laser-on (not shown) and laser-off spectrum is due to the nonlinear area correction of the time-to-mass conversion and a non-zero background.

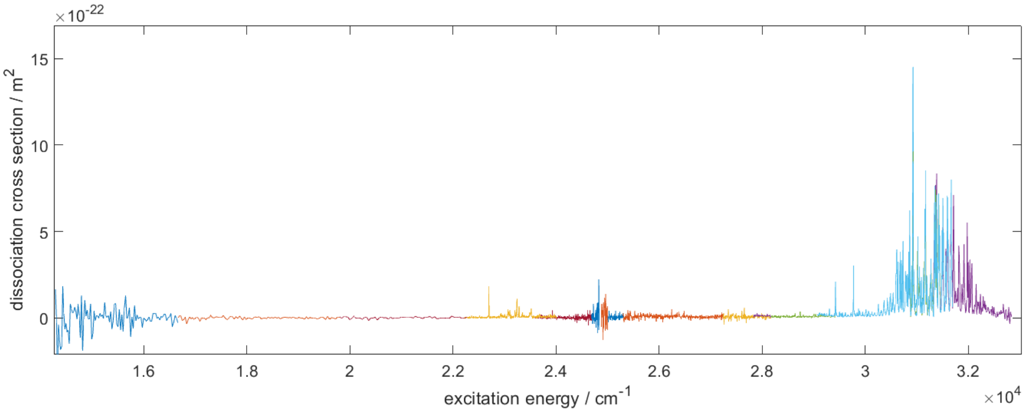

Figure S2. Overview EPD spectrum of Au_2^+ of the whole covered spectral range. A total of 16 different scans are displayed. Regions around 25000 cm^{-1} and below 16000 cm^{-1} have poor signal-to-noise ratio due to low laser power in these ranges.

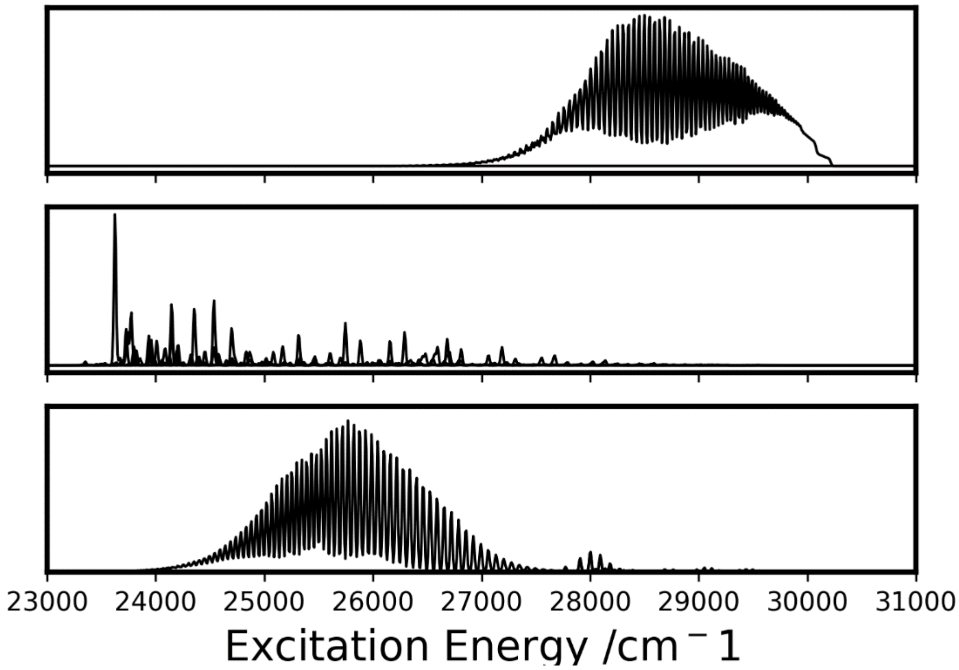

Figure S3. Comparison of the resulting Franck-Condon progressions of the three employed theoretical models. The stick spectra are folded with Gaussian profiles with 10 cm^{-1} FWHM. The simulations were made assuming a temperature of 150 K. Top: MRCI–CASSCF; middle: MRCI–CASSCF–SO; bottom: CAM-B3LYP/aug-cc-pvtz.

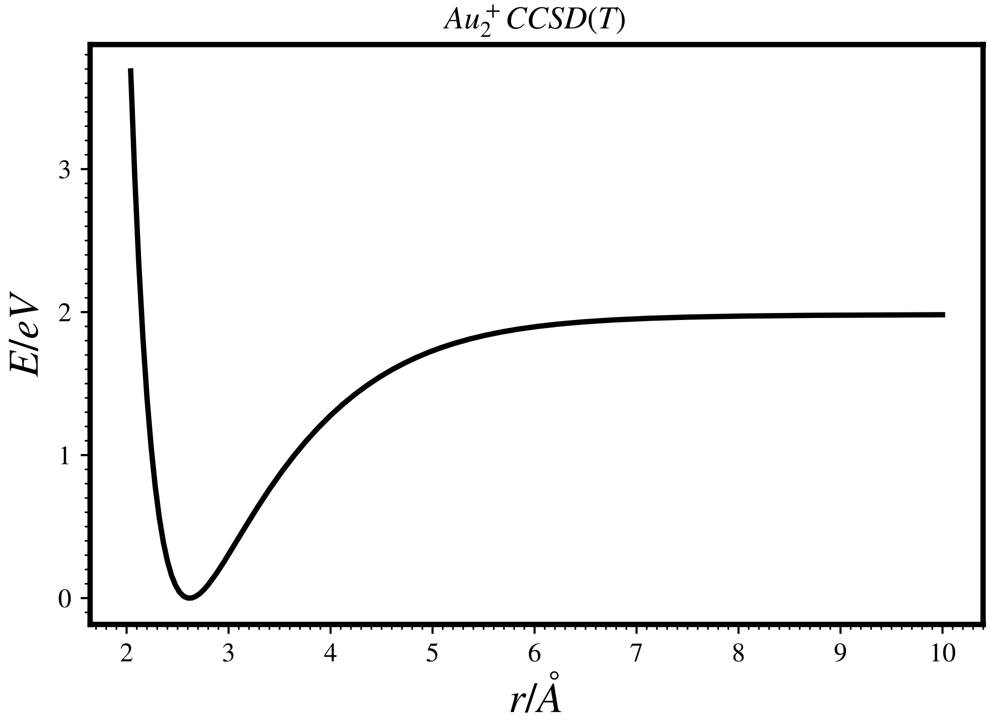

Figure S4. Calculated ground state potential of Au_2^+ at the CCSD(T) level of theory.

Table S1. Positions and intensities of the peaks in the first band system (BS1) measured at 150 K.

peak	position / cm^{-1}	σ / Mb
a1	22520	0.1
a2	22701.5	1.8
a3	22923	0.2
a4	22989	0.1
a5	23012.3	0.2
a6	23065.4	0.5
a7	23081.4	0.35
a8	23105.4	0.46
a9	23204.5	0.45
a10	23245	1.1
a11	23285.6	0.7
a12	23350.8	0.32
a13	23493.5	0.34
a14	23521.1	0.55
a15	23685.5	0.24

Table S2. Positions and intensities of the peaks in the second band system (BS2) measured at 120 K.

peak	position / cm^{-1}	σ / Mb
b1	29308	0.55
b2	29425	2.1
b3	29775	3.0
b4	29873	0.54
b5	29949	0.37
b6	30017	0.48
b7	30075	0.37
b8	30257	0.81
b9	30367	1.0
b10	30432	1.1
b11	30483	1.2
b12	30492	1.4
b13	30544	1.5
b14	30614	4.0
b15	30637	3.3
b16	30680	3.8
b17	30703	3.3
b18	30736	4.4
b19	30783	2.7
b20	30802	2.4

b21	30821	2.5
b22	30845	3.8
b23	30864	6.2
b24	30902	3.0
b25	30931	14.5
b26	31023	4.7
b27	31052	2.9
b28	31093	2.4
b29	31133	2.7
b30	31158	6.3
b31	31168	8.5
b32	31195	2.5
b33	31277	3.4
b34	31311	2.2
b35	31346	7.6
b36	31368	6.5
b37	31387	6.8
b38	31423	7.0
b39	31443	5.4
b40	31492	5.1
b41	31508	6.9
b42	31521	4.9
b43	31564	3.6
b44	31594	7.0
b45	31610	7.0
b46	31670	8.0
b47	31716	7.1
b48	31817	4.2
b49	31878	1.9
b50	31918	4.2
b51	31980	5.5
b52	32000	3.2
b53	32031	3.4
b54	32072	3.2
b55	32154	1.9
b56	32237	1.4
b57	32289	1.3

REFERENCES

1. Förstel, M.; Schewe, W.; Dopfer, O. Optical Spectroscopy of the Au_4^+ Cluster: The Resolved Vibronic Structure Indicates an Unexpected Isomer. *Angew. Chem. Int. Ed.* **2019,** *58* (11), 3356-3360.

2. Förstel, M.; Jaeger, B. K. A.; Schewe, W.; Sporkhorst, P. H. A.; Dopfer, O. Improved tandem mass spectrometer coupled to a laser vaporization cluster ion source. *Rev. Sci. Instrum.* **2017,** *88* (12), 123110.

3. Walther, C.; Dietrich, G.; Kluge, H.-J.; Lindinger, M.; Lützenkirchen, K.; Schweikhard, L.; Ziegler, J. Photofragmentation of metal clusters stored in a Penning trap. *Z. Phys. D: At., Mol. Clusters* **1996,** *38* (1), 51-58.
